# Mapping the Intellectual Landscape of Employee Performance Research: A Bibliometric and Mapping Analysis (2020–2025)

**DOI:** 10.12688/f1000research.165774.1

**Published:** 2025-06-27

**Authors:** Maryadi Maryadi, Hamidah Nayati Utami, Arik Prasetya, Benny Hutahayan

**Affiliations:** 1 Brawijaya University, Malang, East Java, Indonesia

**Keywords:** bibliometric analysis, employee performance, trend topics, research mapping, VOSviewer

## Abstract

**Purpose:**

This study aims to identify research trends and map the academic structure in the field of employee performance over the past five years. Given the increasing complexity of organizations and post-pandemic work dynamics, bibliometric mapping is necessary to understand the evolution of themes, the contributions of key literature, and the future directions of knowledge development.

**Design/Methodology/Approach:**

A total of 2,044 articles published between 2020 and 2025 are collected from the Scopus database. The article selection process adheres to the PRISMA 2020 guidelines to ensure transparency and methodological rigor. Analysis is conducted using Bibliometrix R and VOSviewer, employing performance analysis and science mapping techniques. Visualization results include trending topics, a word cloud, the most globally cited documents, and network visualizations identifying the primary thematic clusters within the literature.

**Findings:**

The findings indicate that research topics are shifting from general issues toward contemporary themes such as digital leadership, work well-being, and organizational adaptation to global changes. Seven primary clusters are identified, reflecting a multidisciplinary approach encompassing structural, psychological, and contextual dimensions. Additionally, articles with high normalized citation scores demonstrate that significant contributions come from practical and cross-sectoral studies.

**Originality/value:**

The novelty of this study lies in mapping the intellectual structure and research trends of employee performance using Bibliometrix R and VOSviewer, focusing on the post-pandemic period (2020-2025). It uniquely employs trending topics and normalized citation analysis to identify emerging themes and influential publications in contemporary research.

## 1. Introduction

In facing an increasingly competitive and uncertain environment, every organization is required to possess the ability to adapt, transform, and continuously strengthen its competitive advantage. ^1^ Organizational success is not solely determined by productivity achievements, but also by the extent to which an organization can manage and leverage its human resources. ^2^ Human resources, represented by individuals who bring energy, creativity, and enthusiasm, play a central role in effectively managing the organization’s operations. ^2, 3^ The presence of employees with exceptional performance becomes a fundamental necessity. High-performing employees are believed to be the primary determinants of organizational success and sustainability in achieving its goals. ^4, 5^ In fact, employee performance is often used as a key indicator to assess the extent to which an organization can optimally carry out its tasks and functions. ^6^ Therefore, improving employee performance is a strategic priority that cannot be overlooked, as it directly contributes to the achievement of the organization’s business objectives. ^7^

Employee performance plays a crucial role in determining the success of an organization. ^8^ Conceptually, employee performance refers to the level of success an individual achieves in completing tasks and their ability to meet previously set targets. ^5, 7, 9^ This performance is reflected through various concrete actions taken by employees in fulfilling their responsibilities and core duties as outlined in their job descriptions. ^6, 10^ Performance serves as a means to achieve better results from organizations, teams, and individuals by understanding and managing performance within the framework of agreed-upon objectives, standards, and attribute requirements. ^11^ Therefore, employee performance can be understood as the result of an individual’s efforts to perform tasks and responsibilities optimally, which ultimately contributes directly to the achievement of the organization’s vision and mission.

In recent years, the topic of employee performance has become a primary focus in various studies within the fields of management and human resources. ^1, 2, 12^ This growing interest indicates that employee performance is not only seen as an indicator of organizational success but also as a strategic variable that influences various aspects of operational efficiency and competitive advantage. Numerous studies have been conducted to uncover the factors influencing employee performance, ranging from leadership, ^13– 15^ motivation, ^16– 18^ work environment, ^19– 21^ to digital transformation. ^22^ However, despite the abundant available literature, there remains a need for efforts to identify key findings and classify the evolving knowledge structure in this field. Mapping the direction and trends of research on employee performance is crucial to understanding research trends, detecting main themes, identifying the most influential publications, and highlighting contemporary topics currently being explored by researchers.

Mapping the direction and trends of research on a particular variable is typically carried out through bibliometric analysis. Several previous studies have conducted bibliometric analyses on the variable of employee performance, such as Mohammad et al., ^23^ Engidaw et al., ^24^ and Iddagoda & Dassanaike. ^25^ The study by Mohammad et al. ^23^ has made significant contributions in mapping the relationships between employee engagement, leadership styles, and employee performance through a bibliometric and systematic review approach. However, this study has several limitations, including the analysis period (2011–2023), which does not fully encompass the recent dynamics of the post-pandemic phase, and the broad thematic focus, which obscures the mapping of the specific academic structure related to employee performance. Additionally, the study by Engidaw et al. ^24^ provides a comprehensive overview of research trends in employee job performance over the past decade. However, it does not specifically address the dynamics of post-pandemic research (2020-2025). The study by Iddagoda & Dassanaike ^25^ offers an initial contribution to mapping trends and dynamics in employee job performance research, but it has limitations that open opportunities for further research development. Its broad temporal scope, without a specific focus on the transformative post-pandemic period (2020–2025), makes it less relevant for capturing contemporary work dynamics.

Therefore, this study aims to fill this gap by exclusively analyzing the trends and intellectual structure of employee performance research through a bibliometric approach using Bibliometrix R and VOSviewer, applied to articles from the Scopus database. The study focuses on the contemporary period (2020-2025), providing fresh and contextual insights into the current development of the academic structure in the field of employee performance, which represents the novelty of this research. This study seeks to identify research trends and map the intellectual structure of the employee performance variable. By identifying research trends and mapping the academic structure, this study opens opportunities to uncover underexplored research gaps and strengthens the theoretical foundation for future studies.

## 2. Literature review

### 2.1 Employee performance

The concept of employee performance has been defined in various ways by different researchers, with complementary approaches. Aliyyah et al. ^16^ state that employee performance is the outcome achieved in terms of quality and quantity according to the responsibilities assigned, reflecting the effectiveness and achievement of individuals in carrying out their tasks. Similarly, Sabuhari et al. ^12^ emphasize that performance encompasses everything done or not done by employees that affects their contribution to the organization, determined by their ability, effort, and organizational support. Ogalo ^26^ reinforces this perspective by highlighting that employee performance reflects an individual’s ability and capacity to complete assigned tasks, and serves as a key indicator of work success or failure. Supardi et al. ^27^ offer a broader perspective, defining performance as behavior that reflects an employee’s actions in carrying out their tasks, where the work outcome is not just the final product, but also the process influenced by various factors such as skills, motivation, and organizational environment. Meanwhile, Atan and Obeng ^28^ stress that performance not only includes observable actions but also the cognitive processes underlying them, as well as the importance of sustainable performance management to align individual work behaviors with the organization’s strategic goals. In a similar vein, Martini et al. ^29^ define employee performance as the outcome of the work or output produced by an employee in carrying out job activities within a specific time frame.

Based on these various perspectives, it can be concluded that employee performance is the level of success achieved by an employee in carrying out their tasks and responsibilities, both in terms of behavior and work outcomes, which are influenced by internal factors (such as ability, motivation, and attitude) and external factors (such as organizational support and performance management systems). This definition encompasses both quantitative and qualitative dimensions, emphasizing the importance of both the process and the results in the context of achieving organizational goals.

### 2.2 Bibliometric analysis

Bibliometric analysis is a quantitative approach used to evaluate, organize, and map scientific literature by utilizing statistical indicators such as publication counts, citation frequency, and relationships between scholarly works. This method was first introduced by Pritchard in 1969 and has since evolved into an essential tool for assessing the scientific structure, identifying research trends, and highlighting the contributions of authors, journals, and institutions that are most influential in a particular field. ^30^ By using software tools such as Bibliometrix in RStudio and VOSviewer, this analysis enables the visualization of conceptual relationships through techniques like co-word analysis, bibliographic coupling, and co-citation analysis, providing a comprehensive overview of the evolution of scientific knowledge. ^31^ In the field of management and human resources research, bibliometrics is not only used to track the development of themes such as employee performance but also to uncover the interconnections between topics, identify seminal works, and provide direction for future research. ^23^ Therefore, bibliometrics functions as a bridge between retrospective mapping and prospective exploration within the scientific research ecosystem.

## 3. Methodology

Before conducting a bibliometric analysis, it is essential to perform a comprehensive and transparent article search to ensure that the data used aligns with the research objectives and follows a rigorous analytical procedure. To achieve this, the PRISMA (Preferred Reporting Items for Systematic Reviews and Meta-Analyses) framework is applied by Page et al. ^32^ PRISMA is a methodological guideline designed to improve the clarity, completeness, and transparency of systematic reviews by providing a structured approach for identifying, screening, and selecting relevant literature. The method includes a 27-item checklist and a flow diagram that guides researchers through four main stages: identification, screening, eligibility, and inclusion. This structure ensures that the final dataset is both relevant to the study and methodologically robust.

The inclusion criteria for this study are as follows:

1. Publication type must be an article;
2. Article must be published in English;
3. Article must be from a peer-reviewed journal;
4. Article must focus on the topic of employee performance; and
5. Publication period must fall between 2020-2025.

Exclusion criteria include:

1. Non-article document types such as books, reviews, and conference papers;
2. Articles not in English;
3. In-press or incomplete documents; and
4. Duplicate entries.

The literature search is conducted using the Scopus database as the primary source. The initial search produces 10,566 documents. An automated filtering process removes documents based on predefined criteria, such as publication year outside 2020 to 2025, non-article document types, in-press status, non-english language, and non-journal sources. This results in 2,172 articles considered eligible for the next step.

The full search strategy, including keywords, boolean operators, and filters applied in Scopus, is available upon request for transparency and reproducibility. The screening stage involves reviewing the titles and abstracts to eliminate irrelevant or duplicate articles. From this process, 58 articles are excluded based on inclusion criteria, which require that the study focus on employee performance and use either quantitative, qualitative, or mixed methods.

**Figure 1.**
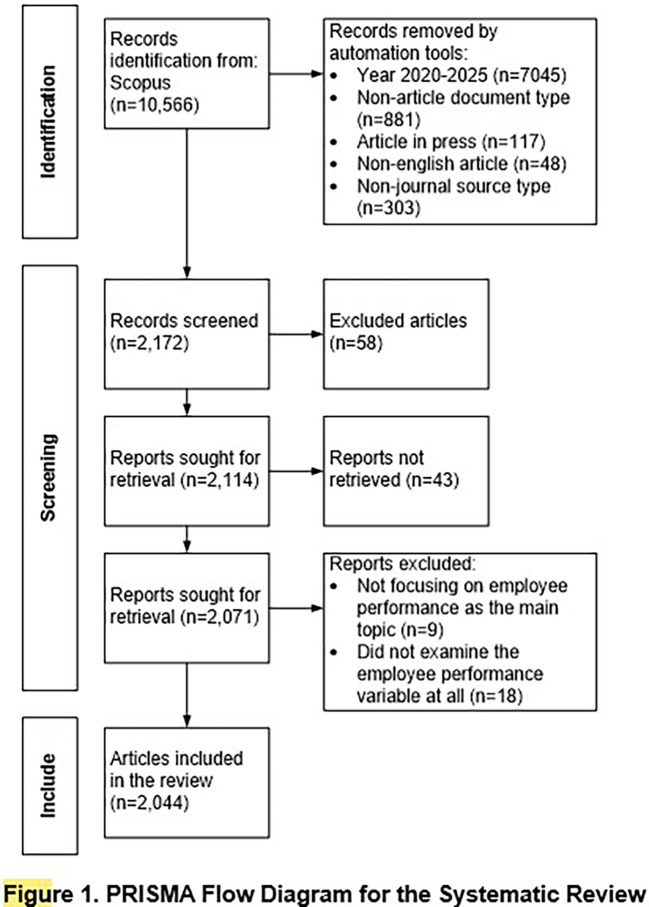
PRISMA flow diagram for the systematic review.

A total of 2,114 articles meets the criteria. In the eligibility stage, the full texts of 2,071 articles are reviewed, and 27 articles are excluded due to incomplete information or failure to address employee performance as a central topic. This leaves 2,044 articles that meet all inclusion criteria. In the final inclusion stage, these 2,044 articles are incorporated into the bibliometric analysis. The entire selection process is summarized in the PRISMA flow diagram presented in Figure 1.

Screening and data extraction are performed independently by two reviewers to reduce bias, with any discrepancies resolved through discussion. No automation tools were used in the screening or extraction process. Building on this rigorous manual process, the systematic steps for conducting bibliometric analysis are outlined based on the framework proposed by Donthu et al. ^33^ as follows:

**Step 1: Define the purpose and scope of the study**

The initial step in this bibliometric analysis is to clearly define the focus and scope of the study. This research aims to identify research trends and map the intellectual structure in the field of employee performance over the past five years (2020-2025). With the topic becoming increasingly relevant in the dynamics of modern organizations, this study explores patterns of scientific contributions, the influence of dominant literature, and the mapping of intellectual relationships between topics in the field. The scope of the study includes 2,044 scholarly articles obtained from the Scopus database, which is considered to have broad coverage and high academic quality in documenting global literature.

**Step 2: Selection of bibliometric analysis techniques**

The second step is to select the appropriate bibliometric analysis techniques, which consist of two main categories:

1. Performance Analysis: This aims to identify the most influential articles through indicators such as publication counts, total citations, and normalized citations. This technique generates outputs like Most Globally Cited Documents, which help identify seminal works and influential themes in the foundation of employee performance theory.
2. Science Mapping: This technique is used to explore thematic relationships between publications through approaches such as co-occurrence and bibliographic coupling. It generates several visual outputs, such as trend topics, word clouds, and network visualizations, providing deep insights into the evolution of topics and the conceptual structure in the analyzed field of study.

**Step 3: Data collection and preparation**

Data is collected from the Scopus database using relevant keywords, such as “employee performance”. The complete search syntax, export settings, and database query filters are documented to ensure transparency. The publication period is limited from 2020 to 2025 to capture the current dynamics of research development. The data is then exported in BibTeX and RIS formats to ensure compatibility with the two primary analysis tools used: Bibliometric R (for statistical and initial graphic analysis) and VOSviewer (for visual network mapping). This step also includes data cleaning, deduplication, and normalization to avoid errors and inconsistencies.

**Step 4: Conducting analysis and reporting findings**

The analysis is performed by combining the functionalities of Bibliometric R and VOSviewer. From Bibliometric, visualizations such as trend topics and word clouds are obtained to detect rapidly developing dominant topics. Through VOSviewer, network mapping is conducted using co-word analysis and bibliographic coupling techniques, identifying seven major clusters within the intellectual structure of employee performance research. The normalized citation results from the top articles are also analyzed to assess the relative influence of scholarly works over time. These findings are presented in the form of graphs, network visuals, and in-depth narrative interpretations, providing a comprehensive overview of the intellectual landscape and contemporary research directions.

The effect measures used include total global citations and normalized citations for comparative purposes. While meta-analysis is not applicable in bibliometric studies, the robustness of interpretations is strengthened through triangulation of performance and science mapping techniques. No subgroup or sensitivity analysis was conducted due to the descriptive nature of bibliometric methods.

**Figure 2.**
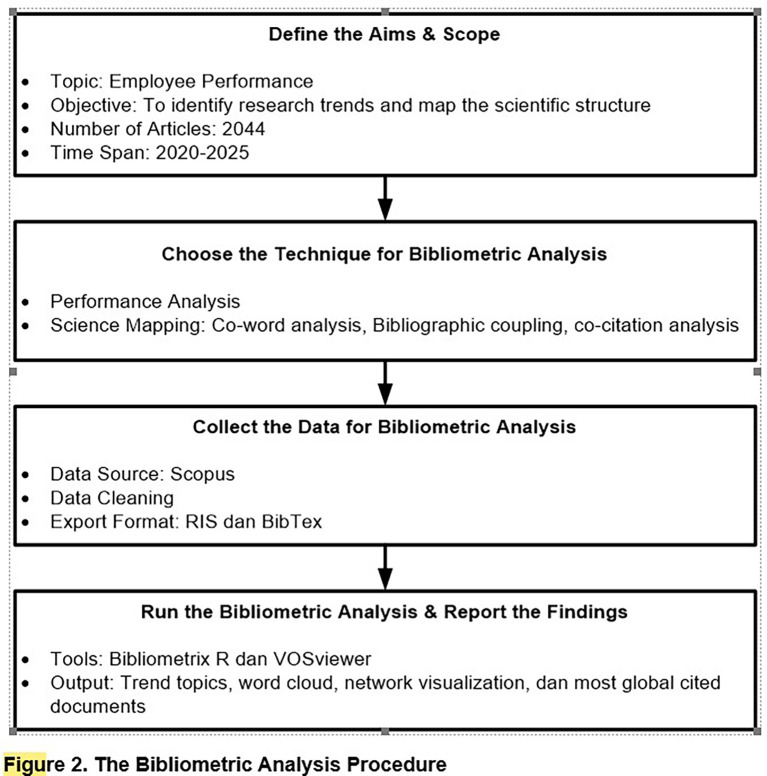
The Bibliometric analysis procedure.

The interpretation process is conducted critically to avoid descriptive bias and produce a rich understanding of the academic dynamics in the field. This process requires researchers to pay careful attention to data validity and the interpretations provided, ensuring that the conclusions drawn are not merely descriptive but also offer analytical insights beneficial to the development of the research area. The bibliometric analysis procedure is graphically depicted in Figure 2.

## 4. Results

### 4.1 Excluded relevant articles

Based on the results of the systematic literature review (SLR) using the PRISMA framework, a total of 2,044 articles meets the inclusion criteria and are used in the bibliometric analysis, while 8,522 articles are excluded. Specifically, 27 articles meet the inclusion criteria but are not included in the analysis for specific reasons. Examples of such articles are presented in Table 1.

**Table 1.** Screened articles excluded from the final bibliometric dataset on employee performance.

No	Authors	Reason for exclusion
1	Armenta-Hernández et al. ^34^	Focuses on improving production processes and lean efficiency rather than employee performance.
2	Hafianti et al. ^35^	Focuses on bibliometric analysis of faculty publication performance rather than employee performance.
3	Sylqa & Neziraj ^36^	Although it examines performance, the article does not explicitly define or empirically measure employee performance.

Although the three articles pass the initial metadata and keyword-based screening, full content analysis reveals that their research substance does not explicitly discuss or measure employee performance as a primary variable or as a structured outcome. The first article ^35^ focuses more on evaluating faculty publication output through bibliometric databases rather than addressing employee performance within an organizational context. The second article ^36^ mentions the term “employee performance”, but does not provide a theoretical definition or sufficient quantitative measurement tools for inclusion in the synthesis. Meanwhile, the third article ^34^ explores a highly technical topic in the context of lean manufacturing and does not focus on the evaluation of individual or group performance. Therefore, these three articles are excluded to ensure that the final synthesis accurately reflects empirical studies aligned with the primary focus of this bibliometric review.

### 4.2 Main information

**Figure 3.**
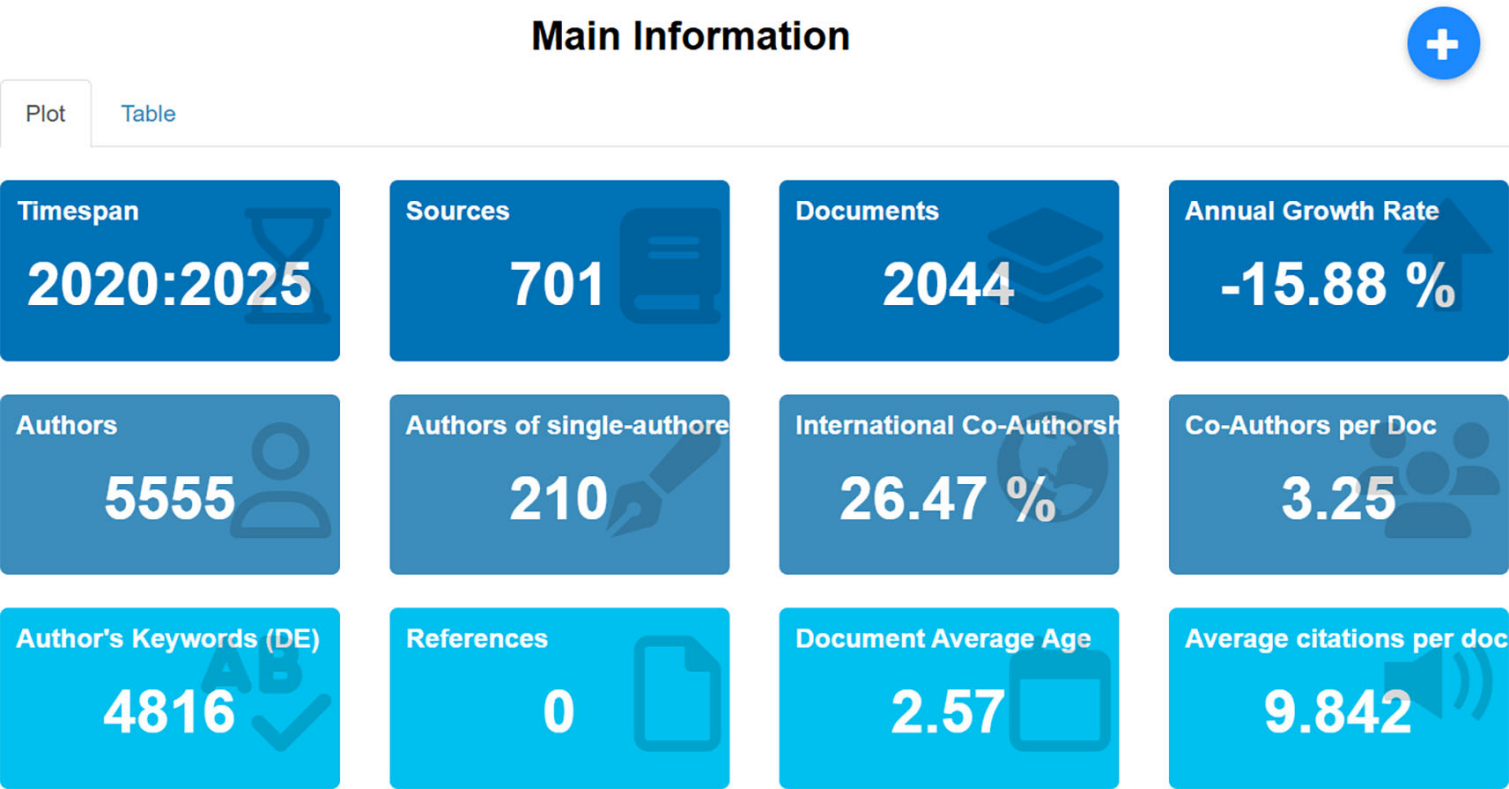
Main information on employee performance articles.

Main Information provides a summary of the general characteristics of the articles analyzed in this study, which includes a total of 2,044 from 10,566 documents related to employee performance published between 2020 and 2025, obtained from the Scopus database. The displayed information includes the number of publication sources, number of authors, level of international collaboration, average document age, frequency of used keywords, and average citations per document. A detailed breakdown of this descriptive information can be seen in Figure 3 below.

Figure 3 presents descriptive information on the bibliometric characteristics of 2,044 scholarly documents analyzed in this study on employee performance published between 2020 and 2025. The total number of publication sources analyzed is 701, with contributions from 5,555 authors, including 210 single authors. This is supported by data showing that the average number of collaborators per article is 3.25, and the proportion of international collaboration is 26.47%, indicating openness and cross-border collaboration in the production of scientific knowledge. However, the annual publication growth rate indicates a negative trend of -15.88%, reflecting either thematic saturation or a shift in research focus toward new, more specific areas. Authors use a total of 4,816 keywords. The average document age is 2.57 years, and the average citation per document is 9.84. Overall, these descriptive data provide a comprehensive overview of the volume, collaboration, temporal dynamics, and scientific quality in the employee performance literature during the reviewed period.

### 4.3 Identifying research trends

**Figure 4.**
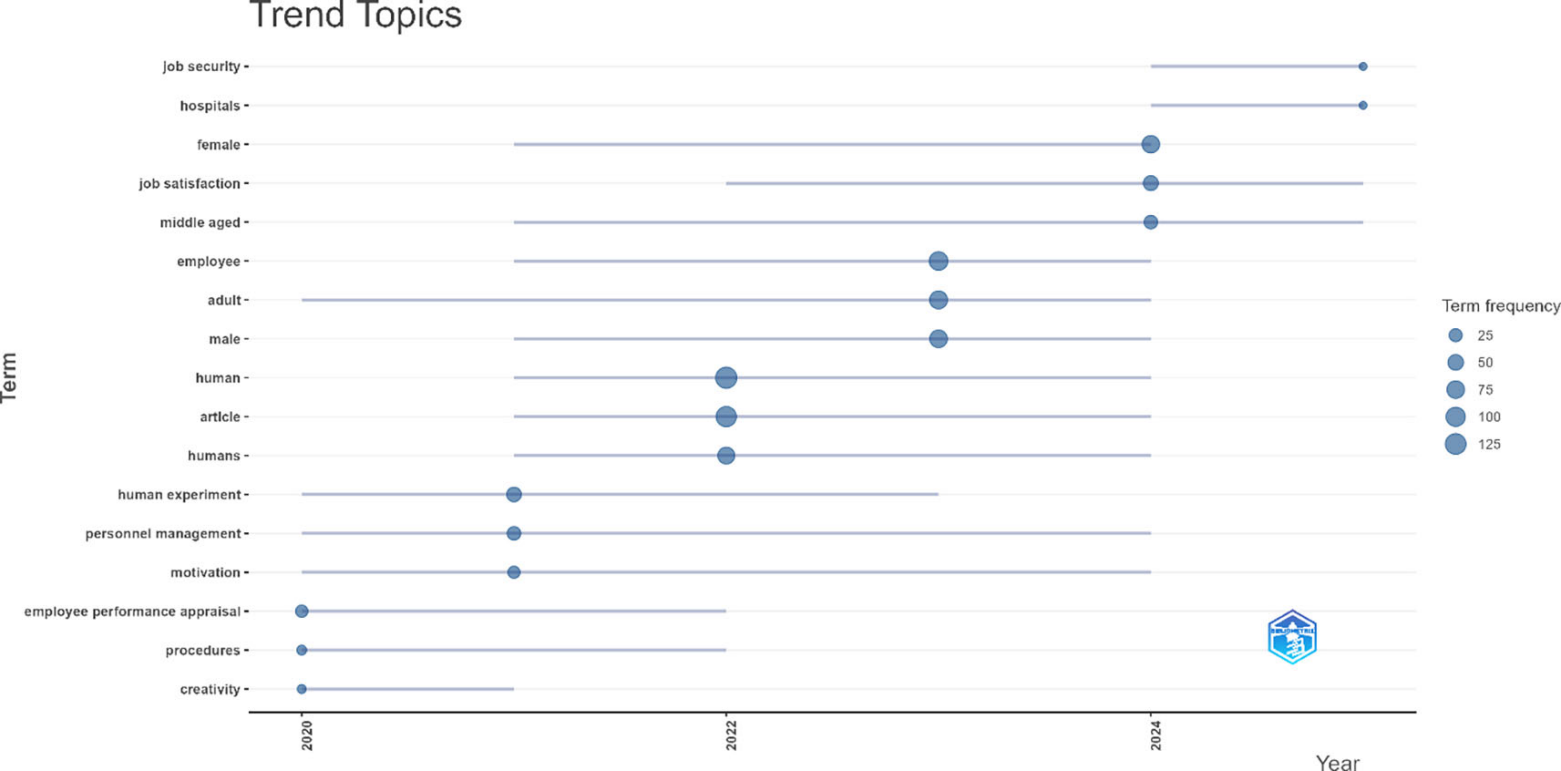
Trend topics in employee performance research.

This section presents the results of identifying research trends in employee performance studies based on bibliometric analysis of publications from 2020 to 2025. The topic trend visualization illustrates the evolution of research themes over time, showing the most frequently appearing topics and their years of emergence. Figure 4 below displays the development and dynamics of the main topics that have been the focus of the literature over the past five years.

Based on the topic trend visualization and frequency presented in Figure 4, generated through the bibliometric analysis of 2,044 articles on employee performance published over the past five years (2020-2025), there is a significant evolution in research themes. As shown in Figure 4, this evolution reflects shifting scholarly attention toward emerging concepts and methodological advancements in the field. Topics such as “human” and “article” have the highest frequency and began emerging from 2021 to 2024. More specific themes such as “personnel management”, “adult”, and “motivation” have consistently appeared from 2020 or 2021 through 2024. Interestingly, topics like “job satisfaction” began to dominate after 2022 and remain relevant until 2025. Meanwhile, terms that appeared in the last years, such as “job security” and “hospitals”, emerged in 2024 with a median year of 2025. Overall, these findings indicate that employee performance research has shifted from fundamental aspects and general populations toward more specific and applicable contemporary and contextual issues.

### 4.4 Detecting key themes/topics

**Figure 5.**
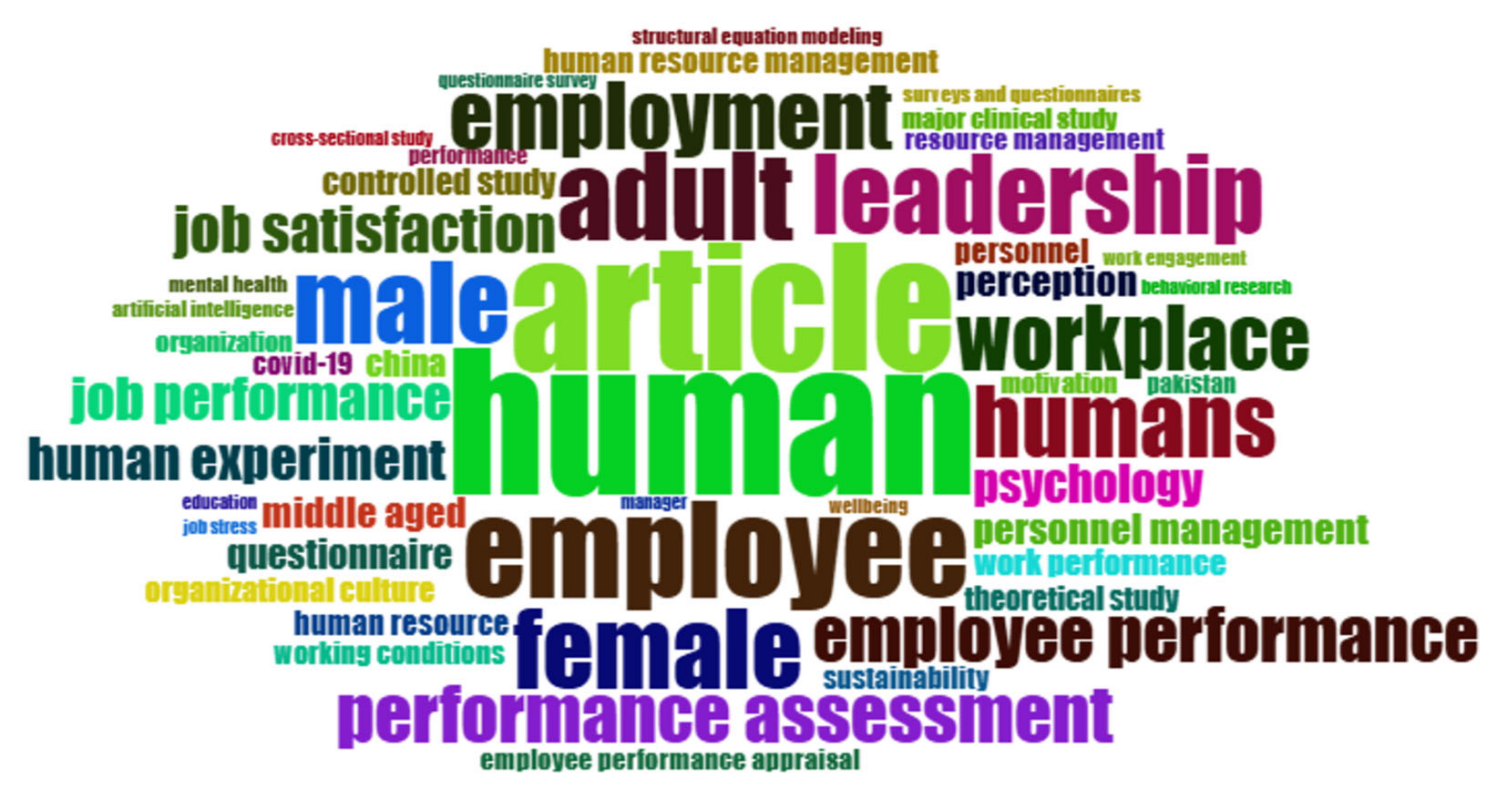
Most frequent words in employee performance themes.

This section aims to detect the main themes or topics most frequently discussed in the employee performance literature through keyword frequency analysis. This identification is carried out to understand the dominant conceptual focus and the terms that consistently appear in publications over the past five years. Figure 5 below presents the visualization of the highest-frequency words that reflect the central themes in related research.

Based on Figure 5, the word cloud and frequency data from the bibliometric analysis of employee performance show that the most common terms are “human” (134), “article” (117), and “employee” (90). This means that most research focuses on people as the main subject and the academic settings where these studies appear. Specific terms such as “job satisfaction” (43), “job performance” (40), “performance assessment” (53), and “employee performance” (50) suggest that researchers widely examine job satisfaction and ways to measure and assess performance. Also, terms like “leadership” (73), “workplace” (59), and “employment” (62) show an increased interest in organizational and management factors affecting performance. Words like “male”, “female”, and “middle-aged” highlight researchers’ attention to demographics in employee performance studies. Therefore, this word cloud provides a clear snapshot showing that recent employee performance research strongly emphasizes organizational factors, leadership roles, assessment methods, and personal characteristics.

The analysis of common words in employee performance studies shows that the terms “human” (134), “article” (117), and “employee” (90) appear most often. Other frequently mentioned terms related to performance include “performance assessment” (53), “employee performance” (50), “job satisfaction” (43), and “job performance” (40). These highlight that research mainly focuses on evaluating and measuring employee performance. Terms such as “male”, “female”, and “adult” show interest in demographics, while “leadership”, “employment”, “workplace”, and “job satisfaction” emphasize organizational factors. Words like “psychology”, “human experiment”, and “perception” suggest that psychological and behavioral viewpoints are important in these studies. Overall, this list clearly illustrates the wide variety of topics and main interests explored in employee performance research in the last five years.

### 4.5 Bibliographic coupling/grouping contemporary themes

**Figure 6.**
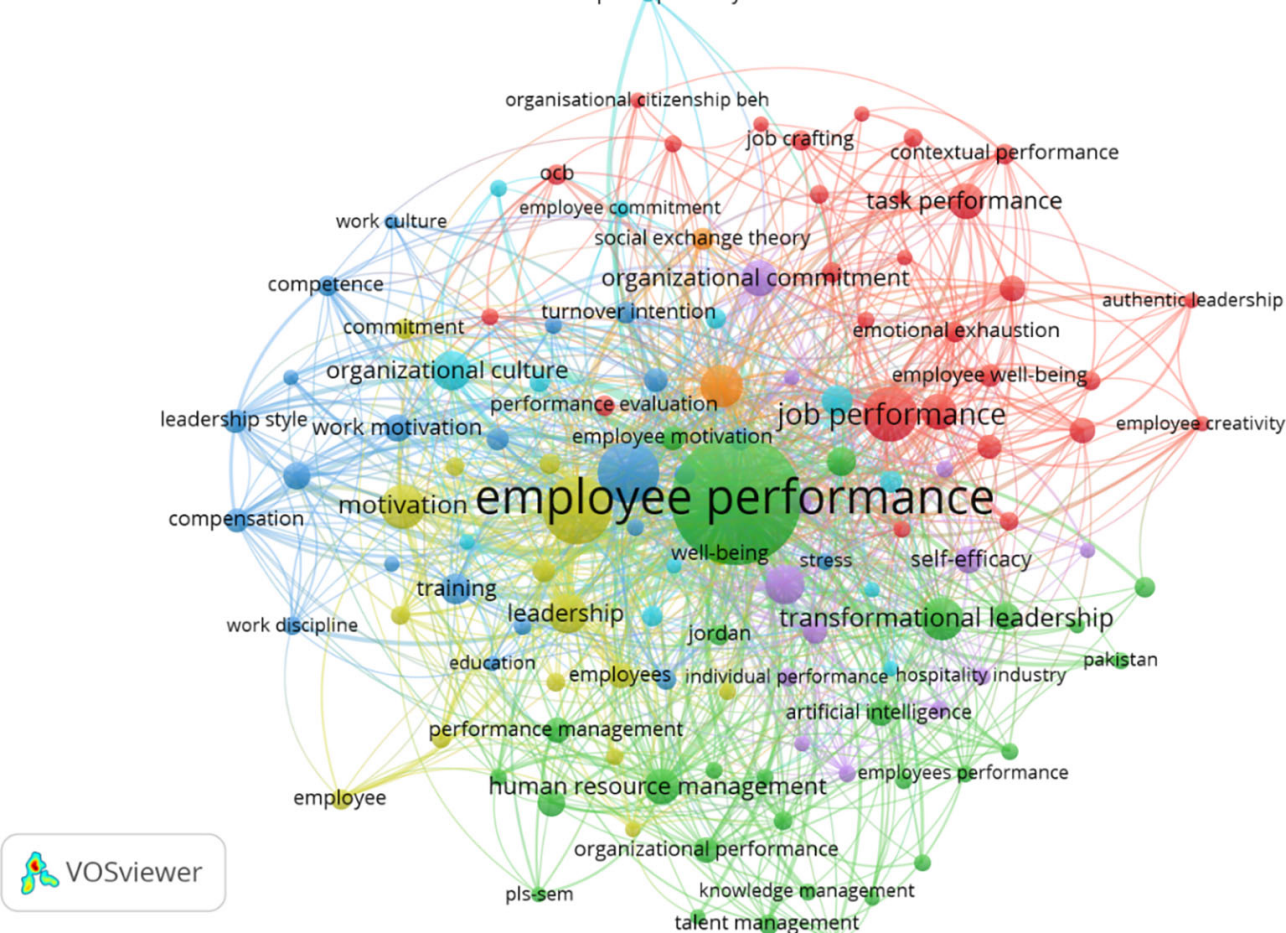
Network visualization of employee performance research.

The network visualization results from the VOSviewer export in employee performance research successfully map seven main clusters, each representing a thematic focus in the literature over the past five years. The VOSviewer output, in the form of a network visualization, is shown in Figure 6.

The mapping of topic interconnections presented in Figure 6 identifies seven main clusters, each representing a distinct thematic focus within the literature on employee performance:

a) The green cluster dominates with managerial issues such as employee performance, transformational leadership, and human resource management, emphasizing the importance of leadership roles and talent management in supporting productivity and innovation.
b) The yellow-green cluster highlights organizational behavior dimensions such as engagement, empowerment, and well-being, emphasizing employees’ emotional and psychological involvement with the work environment.
c) The red cluster focuses on job performance, employee well-being, and ethical leadership aspects, indicating attention to the quality of interpersonal relationships and organizational support.
d) The orange cluster presents a narrower conceptual relationship between employee engagement and social exchange theory, focusing on the theoretical framework of social exchange.
e) The dark blue cluster discusses structural factors such as training, compensation, and work motivation, which are closely related to job satisfaction and productivity.
f ) The light blue cluster combines themes of organizational culture, information technology, and sustainability, demonstrating the integration of organizational culture, digitalization, and sustainability.
g) The purple cluster reflects responses to contextual changes such as Covid-19, digital transformation, and resilience, which highlight the adaptation of organizations and individuals to global disruptions.

Overall, this mapping illustrates the diversity of approaches in employee performance studies, ranging from structural, psychological, to contextual approaches based on contemporary challenges.

### 4.6 Identification of the most influential publications in employee performance research

**Figure 7.**
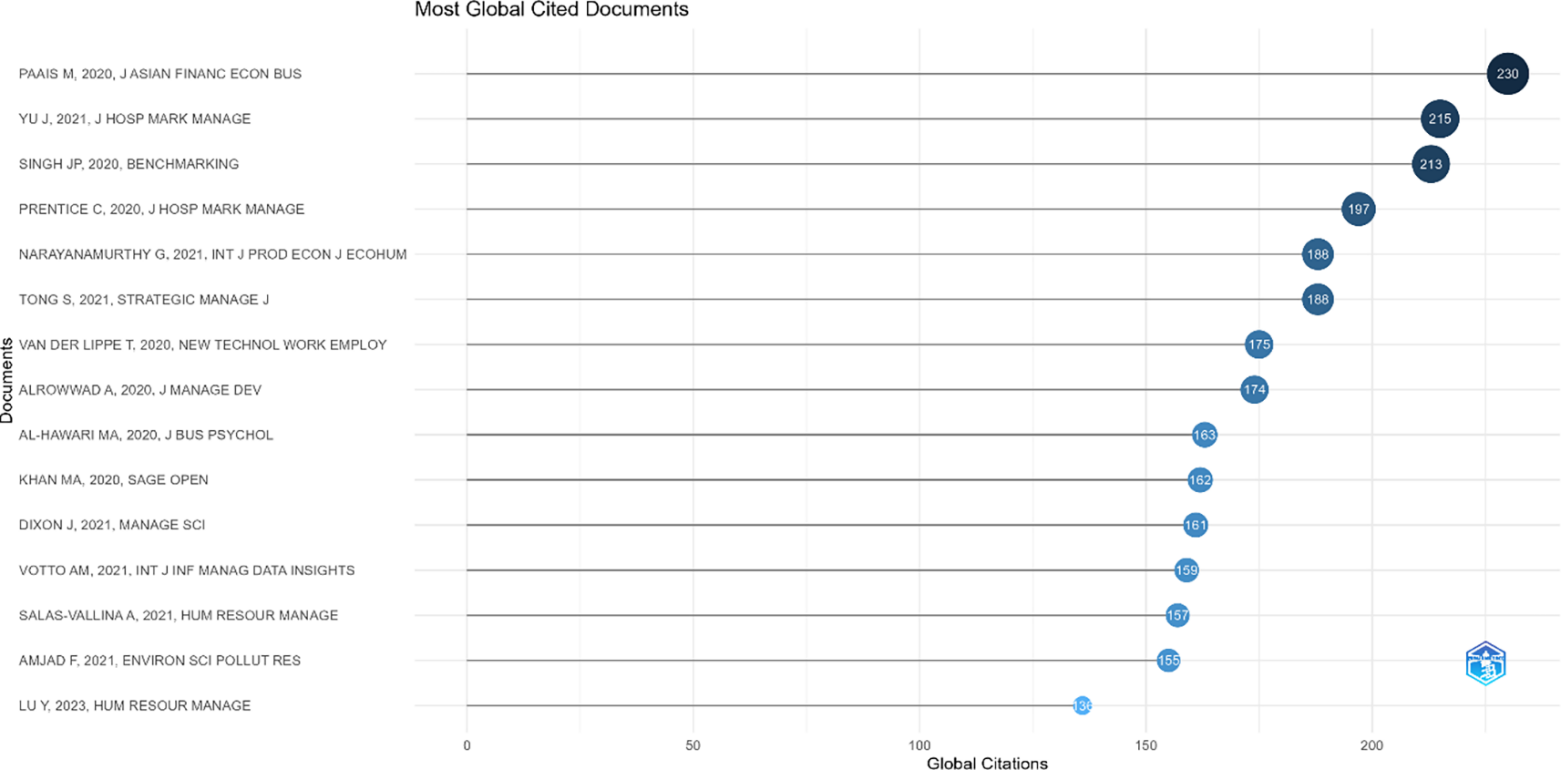
Most global cited documents in employee performance research.

This section presents the results of identifying the most influential publications in employee performance research based on global citation counts. This analysis aims to recognize scholarly works that have made significant contributions in shaping the direction and development of the literature in this field. Figure 7 below displays a list of articles with the highest citation counts, representing the main foundations in the development of employee performance research.

Based on Figure 7, the visualization and tabular data of the Most Globally Cited Documents indicate that the article by Paais and Pattiruhu, ^37^ titled Effect of Motivation, Leadership, and Organizational Culture on Satisfaction and Employee Performance, published in the Journal of Asian Finance, Economics and Business, is the most globally cited work, with 230 citations and an average of 38.33 citations per year. It is followed by the works of Yu et al. ^38^ and Singh et al., ^31^ with 215 and 213 citations, respectively, also indicating a very high level of scientific influence. Other articles, such as Prentice et al. ^39^ and Narayanamurthy & Tortorella, ^40^ also record significant citations, indicating high relevance in the literature related to employee performance.

Table 2 presents the normalized citation data for the three articles with the highest relative academic impact in the field of employee performance. It is evident that the publication by Meneses et al. ^41^ records the highest normalized TC score of 19.51. This article addresses customer satisfaction and dissatisfaction in the context of wine tourism. It is followed by Lu et al. ^42^ with a score of 18.59, which discusses sustainable human resource management practices, employee resilience, and common good values. Next, the article by Chatterjee et al. ^43^ achieves a score of 14.62, focusing on the role of digital leadership capabilities in moderating the relationship between the digital work environment and organizational performance. The high normalized citation values of these three articles indicate that, despite being relatively recent, these publications have made a significant impact in the literature and signal a shift in research attention toward more contemporary, applied themes that are relevant to the challenges of modern organizations.

**Table 2.** Normalized Citation of Employee Performance Research.

No	Author	Year	Title	Normalized TC
1	Meneses, R., Brito, C., Lopes, B., & Correia, R. ^41^	2025	Satisfaction and dissatisfaction in wine tourism: A user-generated content analysis	19.51
2	Lu, Y., Zhang, M. M., Yang, M. M., & Wang, Y. ^42^	2023	Sustainable human resource management practices, employee resilience, and employee outcomes: Toward common good values	18.59
3	Chatterjee, S., Chaudhuri, R., Vrontis, D., & Giovando, G. ^43^	2023	Digital workplace and organization performance: Moderating role of digital leadership capability	14.62

From the perspective of normalized citation, which is a relative measure that allows for comparing academic influence across articles without being distorted by publication time, three articles with the most significant early influence in the field of employee performance are identified. The top-ranked article is by Meneses et al. (2025) (Normalized TC = 19.51), which examines customer satisfaction and dissatisfaction in the context of wine tourism using user-generated content. This study reveals that attributes such as environmental aesthetics, sensory experiences, and staff performance have varying impacts on customer satisfaction and dissatisfaction, supporting two-factor and three-factor theories. This approach offers a new direction in measuring and managing employee performance based on real-time, multidimensional customer perceptions.

## 5. Discussion

### 5.1 Main information

The results from the bibliometric analysis of 2,044 documents related to employee performance published between 2020 and 2025 reveal intriguing dynamics in the landscape of scholarly publications. The relatively high number of sources (701 journals) indicates that this topic has garnered widespread attention across various disciplines, with significant contributions from 5,555 authors. The fact that only 210 publications were written by single authors, with the remainder resulting from collaborations, highlights a strong trend toward collaborative research, both nationally and internationally, as reflected by the 26.47% proportion of international co-authorship. These findings demonstrate that employee performance research is not only multidisciplinary but also involves increasingly active academic networks that transcend borders.

However, the annual publication growth rate decline of -15.88% indicates a trend toward stagnation or reduced interest in general publications in this field, possibly due to thematic saturation or a shift in focus toward more specific and contextual issues, such as digital transformation, work well-being, or organizational sustainability. The relatively young average document age (2.57 years) suggests that the literature used is current and relevant to contemporary developments. Meanwhile, the average citation counts per document, nearing 10, indicates that publications in this field have a strong citation impact, signaling substantial intellectual influence among scholars. The diversity of keywords (4,816) also suggests that employee performance is a broad and multidimensional research area, offering significant opportunities for exploring new topics in future studies. Thus, this descriptive information provides a solid foundation for understanding the structure of scientific production while opening up space for further discussion on the direction and strategic focus of research in the future.

### 5.2 Identifying research trends

The analysis of topic trends in employee performance research from 2020 to 2025 reveals a shift in thematic focus that reflects the adaptive dynamics of scientific inquiry in response to the social context and needs of modern organizations. At the beginning of the period, the emerging themes were still general, such as “human” and “article”, indicating a conceptual focus on individual entities and systematic approaches in scholarly publications. However, as time progressed, researchers’ attention began to shift toward more specific issues, as reflected in the consistent appearance of terms like “personnel management”, “adult”, and “motivation” from 2020-2021 through 2024. These terms show a research trend toward exploring managerial and psychological dimensions related to the management of adult employees and intrinsic motivating factors within organizational contexts.

The most noticeable change appears in the final years of the analysis period, with the emergence of new topics such as “job satisfaction”, “job security”, and “hospitals,” which have become key focuses from 2022 to 2025. The appearance of the term “job satisfaction” as a dominant topic reflects the growing attention to employee psychological well-being as an important indicator of job performance, in line with the increasing uncertainty in the work environment post-pandemic. Meanwhile, topics like “job security” and “hospitals”, which emerge in 2024, indicate that employee performance research is beginning to respond to contemporary issues that are sector-specific and contextual, particularly in the healthcare sector. Overall, the pattern of topic evolution illustrates that employee performance research has shifted from theoretical and descriptive domains to more applied and context-based approaches, simultaneously opening up space for future studies to explore issues relevant to the dynamics of sectors and the ever-changing socio-economic needs.

### 5.3 Detecting key themes/topics

The analysis of the most frequently occurring words in the employee performance literature provides an initial overview of the thematic direction and dominant conceptual focus over the past five years. The words “human” (134 occurrences) and “employee” (90 occurrences) are the most frequently used terms, indicating that research is heavily oriented toward individuals as the central focus within organizations. Terms like “performance assessment” (53), “employee performance” (50), and “job performance” (40) emphasize that performance evaluation remains a core pillar of this research. The repeated use of these terms suggests that the academic discourse not only focuses on the definition of performance itself but also on how it is measured, assessed, and linked to various organizational and psychological variables. Additionally, the appearance of words such as “leadership” and “workplace” reflects that both the work environment and the role of leadership are significant factors associated with the improvement or decline of employee performance.

Interestingly, several terms reflecting demographic and psychological dimensions, such as “male”, “female”, “adult”, and “psychology”, also emerge with high frequency. This suggests that research in this field is not limited to the operational aspects of organizations but also takes into account individual characteristics that influence work behavior. Additionally, the word “article”, appearing 117 times, indicates a connection between studies and literature reviews, as well as a systematic approach to building conceptual frameworks, likely stemming from the use of quantitative and systematic methods such as bibliometrics. Therefore, this data shows that studies on employee performance evolve within a multidisciplinary framework, integrating managerial, psychological, and methodological approaches. These findings serve as an important starting point for understanding how the topic has been shaped and can be directed toward further exploration through thematic mapping and advanced bibliographic analysis.

### 5.4 Grouping contemporary themes

The topic mapping through network visualization in employee performance research identifies seven main clusters that represent the diversity of themes and the contemporary direction of literature development over the past five years. The green cluster emerges as the dominant cluster, focusing on managerial and strategic dimensions, covering themes such as transformational leadership, performance management, human resource management, and talent management. This focus indicates that leadership practices and HRM strategies continue to be central in efforts to enhance employee performance and innovation. On the other hand, the yellow-green and red clusters reflect a significant shift towards psychological and behavioral approaches. Terms such as engagement, well-being, organizational behavior, employee creativity, and psychological capital indicate an increasing focus on non-structural factors related to employee well-being and work experience, which are increasingly recognized as key determinants in achieving optimal performance.

Furthermore, the mapping results also reveal the emergence of contextual themes that reflect the academic response to global challenges and the dynamics of modern organizations. The purple cluster highlights topics such as Covid-19, digital transformation, resilience, and job autonomy, underscoring that issues related to individual resilience and technological adaptation have now become integral components in employee performance research. Meanwhile, the dark blue and light blue clusters focus on structural and work environment elements, such as training, work motivation, organizational culture, and sustainability, indicating that systemic aspects and organizational values continue to play a crucial role in supporting performance. The orange cluster, linking employee engagement with social exchange theory, also signifies the strengthening of the theoretical framework to explain the reciprocal relationship between organizations and employees. Overall, this cluster structure shows that contemporary themes in employee performance research are evolving integratively, combining structural, psychological, social, and contextual aspects, while reflecting a research direction that is increasingly adaptive to the complexities of today’s work environment.

### 5.5 Identification of the most influential publications

The Most Global Cited Documents in employee performance research reveal that works with the highest citation counts tend to address fundamental issues related to the determinants of employee performance within the organizational context. The article by Paais and Pattiruhu, ^37^ which ranks first with 230 citations, highlights the roles of motivation, leadership, and organizational culture in shaping job satisfaction and employee performance. This finding emphasizes that theoretical constructs integrating individual and organizational variables remain highly relevant and serve as key references in the latest literature. Other articles, such as those by Yu et al. ^38^ and Singh et al., ^31^ which each obtained 215 and 213 citations, demonstrate that the focus on aspects such as organizational behavior, work engagement, and performance management strategies remains central to the development of theory and managerial practice. The high citation counts of these articles not only reflect the strength of their empirical and theoretical contributions but also indicate that themes such as leadership, motivation, and work culture have cross-contextual and cross-industrial appeal. Thus, these highly cited works not only shape the conceptual foundation but also serve as markers for the main direction of employee performance research in the global academic landscape.

Analysis of articles with the highest normalized citation values in the employee performance literature reveals a strong tendency towards contextual and responsive approaches to the dynamics of modern organizations. Meneses et al., ^41^ with a normalized TC score of 19.51, ranks at the top through their study examining the determinants of customer satisfaction and dissatisfaction in the tourism sector, specifically wine tourism. The user-generated content-based approach employed in this study emphasizes that customer experience, particularly in relation to service interaction quality and staff performance, has become a key new indicator in assessing employee performance. The high citation value in a short period indicates that research with a sector-specific focus and innovative analytical methods has significant potential to become a reference in interdisciplinary studies, including tourism and human resource management.

Next, Lu et al. ^42^ and Chatterjee et al. ^43^ each obtain normalized TC scores of 18.59 and 14.62, respectively, consistently indicating that sustainability and digitalization are central themes in the latest literature. The study by Lu et al. ^42^ positions sustainable HRM as a key variable that not only impacts employee performance but also resilience and work engagement, both of which are critical elements in sustaining organizational productivity over the long term. On the other hand, Chatterjee et al. ^43^ emphasize the role of digital leadership capabilities in steering and stabilizing technology-based workplace transformation. The focus on digital leadership and adaptability in the face of technological disruption highlights the urgency of aligning organizational practices with changes in the global work environment. Both studies mark a new direction in employee performance research, one that is not solely focused on employee output but also considers systemic conditions, organizational values, and technological readiness as integral parts of the process for achieving sustainable performance.

## 6. Conclusion

The findings from this bibliometric analysis reveal that research on employee performance over the past five years (2020-2025) has undergone significant evolution in terms of thematic focus, theoretical direction, and methodological approaches. Recent research trends have shifted from conventional individual evaluation-based approaches to the integration of contemporary themes such as psychological well-being, digital transformation, sustainable leadership, and adaptation to global disruptions such as the pandemic. The intellectual structure in this field is divided into seven main clusters, reflecting a variety of approaches ranging from structural and managerial dimensions to more complex psychological and contextual aspects. Articles with the highest citation rates and normalized citations indicate that strong contributions come from research emphasizing interdisciplinary approaches and responses to the dynamics of modern organizations. Overall, this research map shows that the study of employee performance has transformed into a landscape that is not only multidimensional but also adaptive to current challenges.

Although research on employee performance has grown significantly, several important gaps remain that can shape future studies. First, few studies look closely at how larger external forces, such as global labor laws, climate change, or political instability, affect employee performance. Second, research in specific fields such as healthcare, education, and creative industries is still limited, even though each has unique performance challenges that need context-specific analysis. Third, mixed-methods and long-term (longitudinal) studies are not used often enough to understand how performance changes over time. Moving forward, future research can aim to build stronger theories, explore a wider range of work settings, and use research designs that better match the complex nature of today’s work environments.

The findings of this study have significant implications for the development of theory and practice in the field of human resource management, particularly related to employee performance. Theoretically, these findings reinforce the need for the integration of organizational behavior theory, occupational psychology, and strategic management in forming a comprehensive and contextual conceptual framework. Theory-based approaches such as the Job Demands-Resources Model, Social Exchange Theory, and Contingency Theory can be used simultaneously to explain the variables influencing performance in various work situations. Practically, these findings provide guidance for practitioners in designing HR policies that not only focus on performance evaluation but also consider dimensions such as well-being, technological adaptation, and work flexibility. Decision-makers in organizations are encouraged to use these findings to create a more supportive, adaptive, and sustainable work environment, in order to enhance overall organizational productivity and competitiveness.

## Ethical approval

This study is a literature review and does not involve human participants, therefore no ethical approval is required.

## Consent to participate

As this research is based on a literature review, consent to participate is not applicable.

## Data Availability

No data associated with this article. Repository name: **Data BIBLIO Employee Performance.** https://doi.org/10.6084/m9.figshare.29115704 ^44^ This project contains the following extended data:
• [Data SLR] (Collection of article data used for literature review) • PRISMA Checklist Data are available under the terms of the Creative Commons Attribution 4.0 International license (CC-BY 4.0).

## References

[1] Laksono AA : The impact learning organization and organization culture to employee performance, mediated by knowledge sharing (Empirical study on Bumitama Agri Ltd.). *Aptisi Transactions on Technopreneurship (ATT).* 2023;5(2):145–156. 10.34306/att.v5i2.294

[2] Idris I Adi KR Soetjipto BE : The mediating role of job satisfaction on compensation, work environment, and employee performance: Evidence from Indonesia. *Entrepreneurship and Sustainability Issues.* 2020;8(2):735–750. 10.9770/jesi.2020.8.2(44)

[3] Rahmawaty A Rokhman W Bawono A : Emotional intelligence, spiritual intelligence and employee performance: The mediating role of communication competence. *International Journal of Business and Society.* 2021;22(2):734–752. 10.33736/ijbs.3754.2021

[4] Al-Husseini S : Intellectual capital dimensions and employee performance: The mediating role of organizational learning. *Cogent Business & Management.* 2023;10(3):2284437. 10.1080/23311975.2023.2284437

[5] Ratnasari SL Sutjahjo G Adam A : The effect of job satisfaction, organizational culture, and leadership on employee performance. *Ann. Trop. Med. Public Health.* 2020;23. 10.36295/ASRO.2020.231329

[6] Kamaruddin MJ Buchdadi AD Wolor CW : The Influence of Digital Leadership and Organizational Culture through Job Satisfaction on Employee Performance of PT. Suara Merdeka Press. *Pakistan Journal of Life and Social Sciences.* 2024;22(1):5519–5531. https://www.pjlss.edu.pk/pdf_files/2024_1/5519-5531.pdf

[7] Masharyono M Arifianti R Sukoco I : Influence of job characteristics, work environment, and engagement on employee performance in Indonesian state-owned enterprises. *Journal of Eastern European and Central Asian Research (JEECAR).* 2023;10(6):853–866. 10.15549/jeecar.v10i6.1506

[8] Ahli R Hilmi MF Abudaqa A : The influence of leadership dynamics and workplace stress on employee performance in the entrepreneurial sector and the moderating role of organizational support. *Aptisi Transactions on Technopreneurship (ATT).* 2024;6(3):300–313. 10.34306/att.v6i3.424

[9] Haryanto B Suprapti AR Taufik A : Moderating role of transformational leadership in the relationship between work conflict and employee performance. *Cogent Business & Management.* 2022;9(1):2105578. 10.1080/23311975.2022.2105578

[10] Alqasa KM Alsulami NY : The impact of flexible work arrangements (FWA) on employees performance in the Saudi education sector. *International Journal of Operations and Quantitative Management.* 2022;28(1):174–192. https://submissions.ijoqm.org/index.php/ijoqm/article/view/46

[11] Zulkifli Z Purwati AA Renaldo N : Employee performance of Sharia Bank in Indonesia: The mediation of organizational innovation and knowledge sharing. *Cogent Business & Management.* 2023;10(3). 10.1080/23311975.2023.2273609

[12] Sabuhari R Sudiro A Irawanto D : The effects of human resource flexibility, employee competency, organizational culture adaptation and job satisfaction on employee performance. *Management Science Letters.* 2020;10(8):1775–1786. https://growingscience.com/beta/msl/3691-the-effects-of-human-resource-flexibility-employee-competency-organizational-culture-adaptation-and-job-satisfaction-on-employee-performance.html

[13] Baig SA Iqbal S Abrar M : Impact of leadership styles on employees’ performance with moderating role of positive psychological capital. *Total Qual. Manag. Bus. Excell.* 2021;32(9-10):1085–1105. 10.1080/14783363.2019.1665011

[14] Liu N Zhang H Zhou J : Unraveling the effect of differential leadership on employee performance: Evidence from China. *Front. Psychol.* 2023;14. 10.3389/fpsyg.2023.1081073 PMC10017960 36935973

[15] Rawashdeh A Elayan M Shamout M : Job satisfaction as a mediator between transformational leadership and employee performance: Evidence from a developing country. *Management Science Letters.* 2020;10(16):3855–3864. https://growingscience.com/beta/msl/4101-job-satisfaction-as-a-mediator-between-transformational-leadership-and-employee-performance-evidence-from-a-developing-country.html

[16] Aliyyah N Prasetyo I Rusdiyanto R : What affects employee performance through work motivation? *Journal of Management Information and Decision Sciences.* 2021;24(1). https://scholar.unair.ac.id/en/publications/what-affects-employee-performance-through-work-motivation

[17] Chien GC Mao I Nergui E : The effect of work motivation on employee performance: Empirical evidence from 4-star hotels in Mongolia. *J. Hum. Resour. Hosp. Tour.* 2020;19(4):473–495. 10.1080/15332845.2020.1763766

[18] Tahiri A Kovaci I Dimoska T : Impact of motivation on employee performance in the hospitality industry. *QUALITY Access to Success.* 2022;23(187):58–64. 10.47750/QAS/23.187.07

[19] Korir J : Psychosocial Work Environment and Employee Performance in Public Hospitality Establishments in Kenya. *African Journal of Hospitality, Tourism and Leisure.* 2023;12(3):899–910. https://www.ajhtl.com/uploads/7/1/6/3/7163688/article_7_12_3_899-910.pdf

[20] Girdwichai L Sriviboon C : Employee motivation and performance: do the work environment and the training matter? *Journal of Security & Sustainability Issues.* 2020; (9):42–54. http://openurl.ebsco.com/EPDB:gcd:11:12591915/detailv2?sid=ebsco:plink:scholar&id=ebsco:gcd:141606530&crl=c&link_origin=scholar.google.com

[21] Srinivas V Prasad KDV Rani R : Relationship between employer branding and employee performance: Mediating and moderating effects of supportive work environment and compensation and benefits. *Humanities and Social Sciences Letters.* 2024;12(4):964–984. 10.18488/73.v12i4.3952

[22] Qiao G Li Y Hong A : The strategic role of digital transformation: Leveraging digital leadership to enhance employee performance and organizational commitment in the digital era. *Systems.* 2024;12(11). 10.3390/systems12110457

[23] Mohammad AM Menhat M Shafi S : Trends in employee performance: A comprehensive review and bibliometric analysis using Scopus and WOS. *SA J. Hum. Resour. Manag.* 2025;23:1–13. 10.4102/sajhrm.v23i0.2887

[24] Engidaw AE Zou W Ning J : Perusing the Contemporary Tendencies in Employees’ Job Performance Studies: A Bibliometric Analysis of Research Trends, 2013–2023. *SAGE Open.* 2025;15(1). 10.1177/21582440251321357

[25] Iddagoda A Dissanayake H Dassanaike H : Reflection of employee job performance through a bibliometric analysis. *Journal of Financial Studies.* 2023;8(8):205–214. 10.55654/JFS.2023.SP.26

[26] Ogalo HS : Impact of Training and development programs on employee performance in the banking sector of bahrain. *International Journal of eBusiness and eGovernment Studies.* 2021;13(2):49–68. https://sobiad.org/menuscript/index.php/ijebeg/article/view/791/80

[27] Supardi C Wibisono MK Ilham RN : The Effect of Creative Self-Efficacy, Training and Development on Employee Performance Through Mediation: Innovative Work Behavior and Moderation: Digital Literacy at the Regional Secretariat of the Riau Archipelago Province. *Quality-Access to Success.* 2024;25(203). 10.47750/QAS/25.203.44

[28] Atan T Obeng HA : An Empirical Exploration of Psychological Well-Being’s Mediating Influence on Work-Life Balance and Employee Performance in Ghanaian Public Hospitals. *Asian Journal of Business and Accounting.* 2024;17:169–205. 10.22452/ajba.vol17no2.5

[29] Martini IAO Gorda AES Gorda AOS : Impact of competence development, on work creativity, employee performance, and competitiveness of woven products. *Cogent Business & Management.* 2024, 2024;11(1). https://www.tandfonline.com/doi/full/10.1080/23311975.2024.2353136

[30] Sundar S Gurupandi M : Exploring Strategic Entrepreneurship Research: A Comprehensive Decadal Bibliometric Analysis. *Int. Rev. Manag. Mark.* 2025;15(1):179–192. 10.32479/irmm.17438

[31] Singh JP Chand PK Mittal A : High-performance work system and organizational citizenship behaviour at the shop floor. *BIJ.* 2020;27(4):1369–1398. 10.1108/BIJ-07-2019-0339

[32] Page MJ McKenzie JE Bossuyt PM : The PRISMA 2020 statement: an updated guideline for reporting systematic reviews. *BMJ.* 2021. 10.1136/bmj.n71 PMC8005924 33782057

[33] Donthu N Kumar S Mukherjee D : How to conduct a bibliometric analysis: An overview and guidelines. *J. Bus. Res.* 2021;133:285–296. 10.1016/j.jbusres.2021.04.070

[34] Armenta-Hernández O Maldonado-Macías A Camacho-Alamilla MDR : The relationship between the burnout syndrome dimensions and body mass index as a moderator variable on obese managers in the Mexican maquiladora industry. *Front. Psychol.* 2021;12. 10.3389/fpsyg.2021.540426 33613371 PMC7889810

[35] Hafianti R Lestari Y Erlinengsih E : Measuring Performance of Padang Panjang Public Hospital in Achieving Its Targets Using the Balanced Scorecard Method. *Media Kesehatan Masyarakat Indonesia.* 2022;18(2):74–82. 10.30597/mkmi.v18i2.19006

[36] Sylqa D Neziraj E : Effects of Performance Appraisal on Remuneration Practices and its Impact on Motivation of Employees in Public and Private Sector Companies in Kosovo. *Journal of Education Culture and Society.* 2022;13(2):341–358. 10.15503/jecs2022.2.341.358

[37] Paais M Pattiruhu JR : Effect of motivation, leadership, and organizational culture on satisfaction and employee performance. *The journal of asian finance, economics and business.* 2020;7(8):577–588. 10.13106/jafeb.2020.vol7.no8.577

[38] Yu J Park J Hyun SS : Impacts of the COVID-19 pandemic on employees’ work stress, well-being, mental health, organizational citizenship behavior, and employee-customer identification. *J. Hosp. Market. Manag.* 2021;30(5):529–548. 10.1080/19368623.2021.1867283

[39] Prentice C Dominique Lopes S Wang X : Emotional intelligence or artificial intelligence–an employee perspective. *J. Hosp. Market. Manag.* 2020;29(4):377–403. 10.1080/19368623.2019.1647124

[40] Narayanamurthy G Tortorella G : Impact of COVID-19 outbreak on employee performance–moderating role of industry 4.0 base technologies. *Int. J. Prod. Econ.* 2021;234:108075. 10.1016/j.ijpe.2021.108075 36569040 PMC9759299

[41] Meneses R Brito C Lopes B : Satisfaction and dissatisfaction in wine tourism: A user-generated content analysis. *Tour. Hosp. Res.* 2025;25(1):120–134. 10.1177/14673584231191989

[42] Lu Y Zhang MM Yang MM : Sustainable human resource management practices, employee resilience, and employee outcomes: Toward common good values. *Hum. Resour. Manag.* 2023;62(3):331–353. 10.1002/hrm.22153

[43] Chatterjee S Chaudhuri R Vrontis D : Digital workplace and organization performance: Moderating role of digital leadership capability. *J. Innov. Knowl.* 2023;8(1):100334. 10.1016/j.jik.2023.100334

[44] Maryadi M Utami HN Prasetya A : Data BIBLIO Employee Performance. *figshare.* 2025. 10.12688/f1000research.165774.2 PMC12508735 41079305

