# Supplementary material for: Mapping the Intellectual Landscape of Employee Performance Research: A Bibliometric and Mapping Analysis (2020–2025)
Source: F1000Res. 2025 Oct 3;14:633. Originally published 2025 Jun 27. [Version 2] doi: 10.12688/f1000research.165774.2 (PMC12508735; doi:10.12688/f1000research.165774.2)
Supplement: Peer review history

# Reviewer report: Reviewer response for version 2

**DOI:** 10.5256/f1000research.188782.r419882

**Published:** 2025-10-08

**Authors:** Solimun Solimun

**Affiliations:** 1 Brawijaya University, Ketawanggede, Jawa Timur, Indonesia

I have carefully reviewed the revised manuscript and the author's detailed responses to the previous comments. The revisions have been addressed thoroughly and thoughtfully, with substantial improvements in structure, methodological clarity, and theoretical grounding. The integration of recent literature strengthens the empirical relevance, and the expanded discussion enhances both academic and practical implications. I therefore have no further comments and recommend the article for indexing in its current form.

Are the rationale for, and objectives of, the Systematic Review clearly stated?

Yes

Is the statistical analysis and its interpretation appropriate?

Partly

If this is a Living Systematic Review, is the ‘living’ method appropriate and is the search schedule clearly defined and justified? (‘Living Systematic Review’ or a variation of this term should be included in the title.)

Yes

Are sufficient details of the methods and analysis provided to allow replication by others?

Yes

Are the conclusions drawn adequately supported by the results presented in the review?

Yes

Reviewer Expertise:

My areas of research are Statistics, Data Science, and Business Management. I have extensive experience conducting Systematic Literature Reciew (SLR), Bibliometric as well as research using Big Data approaches. This background allows me to thoroughly asses the methodoligal rigor, data science application, and theoretical contributions of this article, especially in the context of employee performance research and bibliometric mapping within business and management statistics.

I confirm that I have read this submission and believe that I have an appropriate level of expertise to confirm that it is of an acceptable scientific standard.

# Reviewer report: Reviewer response for version 1

**DOI:** 10.5256/f1000research.182515.r408612

**Published:** 2025-09-24

**Authors:** Solimun Solimun

**Affiliations:** 1 Brawijaya University, Ketawanggede, Jawa Timur, Indonesia

**Methods and Analysis:**

 Clearly presented using PRISMA 2020 and Scopus data. However, limited to a single database making the coverage less comprehensive. Expanding to other databases would improve completeness and replicability.

**Statistical Analysis and Interpretation:**

 The analysis is appropriate for a bibiliometric study. It remains largely descriptive. Advanced techniques conceptual structure mapping should be added for greater depth

Are the rationale for, and objectives of, the Systematic Review clearly stated?

Yes

Is the statistical analysis and its interpretation appropriate?

Partly

If this is a Living Systematic Review, is the ‘living’ method appropriate and is the search schedule clearly defined and justified? (‘Living Systematic Review’ or a variation of this term should be included in the title.)

Yes

Are sufficient details of the methods and analysis provided to allow replication by others?

Yes

Are the conclusions drawn adequately supported by the results presented in the review?

Yes

Reviewer Expertise:

My areas of research are Statistics, Data Science, and Business Management. I have extensive experience conducting Systematic Literature Reciew (SLR), Bibliometric as well as research using Big Data approaches. This background allows me to thoroughly asses the methodoligal rigor, data science application, and theoretical contributions of this article, especially in the context of employee performance research and bibliometric mapping within business and management statistics.

I confirm that I have read this submission and believe that I have an appropriate level of expertise to confirm that it is of an acceptable scientific standard, however I have significant reservations, as outlined above.

# Response

**Published:** 2025-09-25

**Authors:** Maryadi Maryadi

**Affiliations:** Brawijaya University, Malang, East Java, Indonesia

Dear Reviewer,

 Thank you for your constructive feedback. In response, we clarify that this study uses Scopus as the primary database. We have therefore added an explanation in the limitation section to emphasize that the scope is restricted to Scopus-indexed articles. Furthermore, beyond the descriptive bibliometric analysis, we now apply thematic analysis to map and interpret the conceptual structure of the literature. This addition enhances the depth of the study by not only describing publication trends but also identifying thematic clusters and conceptual relationships more comprehensively.

 Best regards,

 Maryadi (on behalf of the authors)

# Reviewer report: Reviewer response for version 1

**DOI:** 10.5256/f1000research.182515.r411193

**Published:** 2025-09-18

**Authors:** Sudha Vemaraju

**Affiliations:** 1 GITAM University, Visakhapatnam, Andhra Pradesh, India

1. The article sets a clear context, establishing why a detailed examination of employee performance research is essential. Authors clearly stated its vital relevance by anchoring the analysis in the unprecedented post-pandemic transformation of work. Further the study highlights that existing performance paradigms need to revisted, post pandemic justifying a timely bibliometric mapping to uncover the new research priorities and thematic clusters shaping the future of employee performance. The objectives of the review are then presented in a precise manner, and clearly stated why the contribution is significant.

 2. This study presents a clear methodological framework for bibliometric analysis. The authors clearly mentioned the Scopus as the data source and the time frame (2020–2025), ensuring transparency and ease of replication. PRISMA 2020 guidelines used in this study adds to the rigor and objectivity of article selection, while the adoption of Bibliometrix (R) and VOSviewer further adds credibility to the analysis as they are the leading tools in this research. The outlined approach, from performance evaluation to PRISMA, exclusion of articles, identifying research trends, detecting key themes/topics, grouping contemporary themes, identification of the most influential publications in employee performance research, normalized citation provides a logical and well-structured roadmap. Overall, the methodology is robust, systematic, and sufficiently detailed to serve as a reliable model for future research.

 3. The statistical analysis adopted is well-suited to bibliometric study. The integration of Bibliometrix R for performance analytics and trend visualization with VOSviewer for co-word analysis and bibliographic coupling reflects a standard approach. The seven dominant thematic clusters, normalized citation analysis enhances validity by controlling for temporal and disciplinary biases. This combination ensures a robust synthesis of the intellectual structure and emerging trajectories in employee performance research.

 4. The conclusions are firmly rooted in the empirical results. The seven thematic clusters identified through network mapping, alongside trend and citation analyses, provide direct and compelling evidence for the stated claims about the field's intellectual structure and evolution.

 5. Key Deficits for Living Systematic Review (LSR) Classification:

 Title: The absence of the term "Living Systematic Review" or a clear variant. The specific time-period end date (2025) further states that it is not a living, review.

 Absence of a Defined Search Update Schedule:  The publicly stated schedule for periodic updates is missing. Frequency: How often will the search be repeated (e.g., monthly, quarterly, biannually)?

 Update Triggers: What criteria will trigger an update (e.g., a volume of new publications, emergence of a landmark study)?

 Stopping Criteria: Under what conditions would the "living" status be suspended or ended?

 Absence of Justification for a Living Approach

 No Protocol for Integrating New Evidence: There is no mention of the operational processes for managing updates like:

 How new records will be screened and integrated into the existing analysis. How the bibliometric maps (clusters, networks, trends) will be revised and re-interpreted.

 How version control will be managed and previous iterations archived.

 Conclusion and Recommendation:

 In its present form, this study is a well-executed  bibliometric review with a defined endpoint. To be transformed to an LSR framework, the authors must:

 Amend the title to include "Living Systematic Review."

 Present a detailed protocol outlining the update schedule, justification, and methods for evidence integration by securing a commitment for the long-term resources. Without these critical components, labeling it as a Living Systematic Review would be inappropriate.

 However, the current title is apt for the article and need not be transformed to LSR framework.

Are the rationale for, and objectives of, the Systematic Review clearly stated?

Yes

Is the statistical analysis and its interpretation appropriate?

Yes

If this is a Living Systematic Review, is the ‘living’ method appropriate and is the search schedule clearly defined and justified? (‘Living Systematic Review’ or a variation of this term should be included in the title.)

No

Are sufficient details of the methods and analysis provided to allow replication by others?

Yes

Are the conclusions drawn adequately supported by the results presented in the review?

Yes

Reviewer Expertise:

Marketing, ICTs and Development, AI, ESG, Entrepreneurship, Sustainability, Retail Management, Consumer Behavior, Customer Relationship Management.

I confirm that I have read this submission and believe that I have an appropriate level of expertise to confirm that it is of an acceptable scientific standard.

# Response

**Published:** 2025-09-25

**Authors:** Maryadi Maryadi

**Affiliations:** Brawijaya University, Malang, East Java, Indonesia

Dear Reviewer,

 We sincerely thank you for your valuable feedback and greatly appreciate the constructive suggestions you provide. We would like to clarify that this study is a Systematic Literature Review (SLR) and not a Living Systematic Review (LSR). Accordingly, we add a section on the study’s limitations to clearly emphasize its defined endpoint and scope.

 Best regards,

 Maryadi (on behalf of the authors)
